# Comparing Self-Report Measures of Internalized Weight Stigma: The Weight Self-Stigma Questionnaire versus the Weight Bias Internalization Scale

**DOI:** 10.1371/journal.pone.0165566

**Published:** 2016-10-28

**Authors:** Claudia Hübner, Ricarda Schmidt, Janine Selle, Hinrich Köhler, Astrid Müller, Martina de Zwaan, Anja Hilbert

**Affiliations:** 1 Leipzig University Medical Center, Integrated Research and Treatment Center AdiposityDiseases, Medical Psychology and Medical Sociology, Department of Psychosomatic Medicine and Psychotherapy, Leipzig, Germany; 2 Department of Psychosomatic Medicine and Psychotherapy, Hannover Medical School, Hannover, Germany; 3 Department of Surgery, Herzogin Elisabeth Hospital, Braunschweig, Germany; Universiti Sains Malaysia, MALAYSIA

## Abstract

**Background:**

Internalized weight stigma has gained growing interest due to its association with multiple health impairments in individuals with obesity. Especially high internalized weight stigma is reported by individuals undergoing bariatric surgery. For assessing this concept, two different self-report questionnaires are available, but have never been compared: the Weight Self-Stigma Questionnaire (WSSQ) and the Weight Bias Internalization Scale (WBIS). The purpose of the present study was to provide and to compare reliability, convergent validity with and predictive values for psychosocial health outcomes for the WSSQ and WBIS.

**Methods:**

The WSSQ and the WBIS were used to assess internalized weight stigma in *N* = 78 prebariatric surgery patients. Further, body mass index (BMI) was assessed and body image, quality of life, self-esteem, depression, and anxiety were measured by well-established self-report questionnaires. Reliability, correlation, and regression analyses were conducted.

**Results:**

Internal consistency of the WSSQ was acceptable, while good internal consistency was found for the WBIS. Both measures were significantly correlated with each other and body image. While only the WSSQ was correlated with overweight preoccupation, only the WBIS was correlated with appearance evaluation. Both measures were not associated with BMI. However, correlation coefficients did not differ between the WSSQ and the WBIS for all associations with validity measures. Further, both measures significantly predicted quality of life, self-esteem, depression, and anxiety, while the WBIS explained significantly more variance than the WSSQ total score for self-esteem.

**Conclusions:**

Findings indicate the WSSQ and the WBIS to be reliable and valid assessments of internalized weight stigma in prebariatric surgery patients, although the WBIS showed marginally more favorable results than the WSSQ. For both measures, longitudinal studies on stability and predictive validity are warranted, for example, for weight-related and psychosocial outcomes.

## Introduction

Internalization of weight stigma in individuals with overweight and obesity has gained growing clinical and research interest due to its significant associations with multiple health impairments. Two different self-report questionnaires are internationally available for assessing this concept, but have never been compared: the Weight Self-Stigma Questionnaire [1] and the Weight Bias Internalization Scale [2]. Therefore, the present study sought to compare their psychometric properties and predictive values for health outcomes.

During the last decades, the prevalence of weight stigmatization has substantially increased [3,4]. Weight stigmatization characterized by weight-related negative stereotypes, prejudice, and discrimination [5] is omnipresent. Individuals with overweight and obesity are exposed to weight stigmatization in many domains of life, for example, in employment, in educational and health care settings, in the media as well as in interpersonal relationships [6]. Research provides strong empirical evidence that experiencing weight stigma is associated with greater psychosocial impairment [7–9] and is more likely with increasing body mass index (BMI, kg/m^2^; [10]).

As a specific consequence of weight stigma experiences, individuals with overweight and obesity tend to internalize public weight stigma, applying the predominant, negative weight stereotypes to the own person [2]. More stigmatization of bariatric surgery patients relative to individuals with obesity in conservative weight loss treatment was found after standardizing for BMI and achieved weight loss, potentially because less personal responsibility for the weight loss was attributed to patients pursuing this weight loss approach [11–13]. Relatedly, internalized weight stigma was greater in the specific subsample of individuals undergoing bariatric surgery [14] compared to the population of individuals with overweight and obesity [15]. Interestingly, research produced heterogeneous results with regard to the association of internalized weight stigma and BMI: while internalized weight stigma was positively correlated with BMI in one study [1], there was no association between internalized weight stigma and BMI in other studies [2,16]. Previous studies yielded associations between internalized weight stigma and higher levels of psychopathology (e.g., depression, anxiety, eating disorder psychopathology, body image concern, lower self-esteem, and quality of life), and poorer physical and mental health in individuals with overweight and obesity in general as well as in the specific subsample of prebariatric surgery patients [2,14,15,17–23]. The explanatory power of internalized weight stigma on psychosocial aspects (e.g., depression, body image, exercise behavior) exceeded that of BMI, depression, self-esteem, own stigmatizing attitudes, and own weight stigma experiences [2,8,15,22]. Additionally, internalized weight stigma predicted poorer weight loss outcome in bariatric patients 12 months after surgery [24] as well as in individuals with overweight and obesity six months after conservative weight loss treatment [25]. Altogether, previous findings underscore the relevance of assessing this concept for improving treatment outcomes of individuals with high levels of internalized weight stigma.

The most common assessment avenues of internalized weight stigma rely on self-reports with two questionnaires being available: the Weight Self-Stigma Questionnaire (WSSQ) [1] and the Weight Bias Internalization Scale (WBIS) [2]. The reliable WSSQ was validated in individuals with overweight and obesity and considers internalized weight stigma as a construct with two distinct factors–self-devaluation and fear of enacted stigma–each assessed by six items [1]. In contrast, the WBIS originally contains 11 items assessing internalized weight stigma as a single construct. It is internationally well-established with reliable, valid population norms being available [15]. However, a comparison between the WSSQ and the WBIS with regard to reliability, convergent validity with as well as predictive values for health outcomes has not been conducted until yet.

The present study aimed to investigate item statistics of the WSSQ, its reliability, convergent validity, and predictive values for diverse psychosocial outcomes (i.e., quality of life, self-esteem, depression, anxiety) in a German prebariatric surgery sample as individuals undergoing bariatric surgery report especially high internalized weight stigma [14,15]. Further, reliability, convergent validity, and predictive values were compared between the WSSQ and the WBIS as well as between the WSSQ subscales. It was hypothesized that the WSSQ, its subscales, and the WBIS would show good reliability. In addition, the WSSQ, its subscales, and the WBIS were assumed to be positively associated with each other and significantly associated with negative body image. Due to inconsistent findings, no assumptions were made with regard to the association of the WSSQ, its subscales, and the WBIS with BMI. It was further hypothesized that the WSSQ, its subscales, and the WBIS would predict poorer quality of life and self-esteem as well as greater depression and anxiety.

## Materials and Methods

### Study Design and Participants

This study was part of a larger project that investigated the impact of body contouring surgery after bariatric surgery on psychosocial aspects. It was approved by the Institutional Review Board of Hannover Medical School and was conducted according to the principles expressed in the Declaration of Helsinki. The larger project is described in detail elsewhere [26]. The present cross-sectional study included all data of a subsample consisting of *N* = 78 patients prior bariatric surgery recruited between September 2013 and May 2014 in the larger project through the Department of Psychosomatic Medicine and Psychotherapy at Hannover Medical School. All participants provided written informed consent prior to study participation. Paper- and pencil-based data collection proceeded independently from psychological evaluation and patients were informed that the study data would not be shared with clinicians.

### Questionnaire Translation

The original English WSSQ [1] was translated by the German-speaking first author into German. This translated version was reviewed with regard to practicability by the workgroup comprising researchers with multi-annual expertise in the field of obesity resulting in minor changes with respect to German expression and syntax. Hence, the corrected German version of the WSSQ was back-translated by a blinded certified licensed translator. Subsequently, a congruence check of the back-translated version was conducted without detecting any incongruence.

### Measures

#### Weight Self-Stigma Questionnaire (WSSQ)

**The** WSSQ assesses two aspects of internalized weight stigma: self-devaluation (e.g., “I caused my weight problems”) and fear of enacted stigma (e.g., “I feel insecure about others’ opinions of me”) [1]. Each subscale contained six items rated on a 5-point Likert scale ranging from 1 = *completely disagree* to 5 = *completely agree*. Sum scores for each subscale as well as a total sum score were computed with higher scores indicating greater internalized weight stigma. Good validity and reliability have been documented for the original English version with internal consistencies of α = 0.88 (WSSQ total score), α = 0.87 (WSSQ fear of enacted stigma), and α = 0.81 (WSSQ self-devaluation), respectively [1].

#### Weight Bias Internalization Scale (WBIS)

Each item of the 11-item version of the WBIS ([2,15]; e.g., “I feel anxious about being overweight because of what people might think of me”) was rated on a 7-point Likert scale from 1 = *strongly disagree* to 7 = *strongly agree*. As recent psychometric analysis of the WBIS recommended removal of item 1 due to insufficient item-total correlations [15], a mean score was computed of the remaining 10 items with higher scores indicating greater internalized weight stigma. The 10-item version of the German WBIS has shown good validity and excellent reliability with an internal consistency of α = 0.91 [15].

#### Multidimensional Body-Self Relations Questionnaire–Appearance Scales (MBSRQ-AS)

To assess participants’ body image, three subscales of the MBSRQ-AS [27,28] were used: overweight preoccupation (four items), appearance evaluation (seven items), and appearance orientation (12 items), rated on a 5-point Likert scale from 1 = *definitely disagree*/*never* to 5 = *definitely agree/very often*. Overweight preoccupation includes the concepts fat anxiety, weight vigilance, dieting, and restrained eating. While appearance evaluation assesses appraisal of the own appearance, appearance orientation focuses on cognitive-behavioral investment in the own appearance indicated by its importance in thoughts and behavior. For each subscale, a mean score was computed with higher scores indicating more overweight preoccupation, better appearance evaluation, and more appearance orientation, respectively. Good validity and acceptable to excellent reliability of the mentioned German subscales have been shown in a sample consisting of individuals with eating disorder psychopathology and healthy controls: internal consistencies were α = 0.78 (overweight preoccupation), α = 0.90 (appearance evaluation), and α = 0.82 (appearance orientation), respectively [28]. In this study’s sample, internal consistencies were α = 0.44 (overweight preoccupation), α = 0.56 (appearance evaluation), and α = 0.83 (appearance orientation), respectively.

#### Impact of Weight on Quality of Life–Lite (IWQOL-Lite) Questionnaire

The IWQOL-Lite [29,30] was used to assess quality of life in obesity. It contains five subscales (physical function, self-esteem, sexual life, public distress, work) with a total of 31 items rated on a 5-point Likert scale from 1 = *never true* to 5 = *always true*. Good validity and excellent reliability of the German IWQOL-Lite have been shown in previous research with internal consistencies of α = 0.97 (IWQOL-Lite total score) and α = 0.94 (IWQOL-Lite self-esteem), respectively [30]. In this study, we computed a total score as well as a sum score for the 7-item subscale self-esteem, with higher scores indicating poorer quality of life and self-esteem. Internal consistency in this study’s sample was α = .94 for the total score and α = .92 for the self-esteem subscale.

#### Patient Health Questionnaire–depression scale (PHQ-9)

The German PHQ-9 [31] was used to measure depression severity. Each of the nine items was rated on a 4-point Likert scale from 0 = *not at all* to 3 = *nearly every day*. A sum score was computed with higher scores indicating more severe depression. The PHQ-9 has shown good validity and reliability in primary care patients with an internal consistency of α = .89 [31]. Internal consistency in this study’s sample was α = .81.

#### 7-item Generalized Anxiety Disorder Scale (GAD-7)

To assess participants’ symptom severity of generalized anxiety disorder, we administered the GAD-7 [32,33]. Each of the seven items was rated on a 4-point Likert scale ranging from 0 = *not at all* to 3 = *nearly every day*. A sum score was computed with higher scores indicating more severe symptoms. The German GAD-7 has shown good validity and reliability in the general population with an internal consistency of α = .89 [33]. In this study’s sample, internal consistency was α = .88.

### Data Analytic Plan

Computing total or subscale scores, workgroup internal standard was used: missing values were replaced by participant’s mean of the scale and subscale, respectively, if there were less than 25% missing values per participant and per scale and subscale, respectively. Hence, no total or subscale scores were computed, if the proportion of missing values per participant and per scale and subscale, respectively, was 25% or higher. Regarding item analysis of the WSSQ, evaluation of skewness and kurtosis were based on the ratio of item’s skewness with item’s skewness standard error and item’s kurtosis with item’s kurtosis standard error. Values greater than ± 1.96 suggest significant differences from normality with regard to skewness and kurtosis, respectively [34]. For computing item difficulty, WSSQ items were first recoded to 0 to 4, then difficulty indices were estimated as *p*_*m*_ = (sum of recoded item scores * 100)/ (*N* * maximal item score) [35]. Pearson’s *r* was calculated for corrected item-total-correlations and due to non-normality, Spearman rank order correlations were used for correlation of the item with the corresponding and non-corresponding subscale. Cronbach’s α was computed for evaluation of internal consistency of WSSQ, its subscales, and the WBIS. Pearson correlation analyses of the WSSQ and its subscales with the WBIS and MBSRQ-AS subscales and, in case of non-normality, Spearman rank order correlation analyses were used to evaluate convergent validity.

Further, separate linear regression analyses were conducted to examine the predictive value of the WSSQ, its subscales, and the WBIS on psychosocial outcome measures of the IWQOL-Lite, IWQOL-Lite self-esteem, PHQ-9, and GAD-7. Interpretation of correlation coefficients (small: ≥ .10 and < .30; medium: ≥ .30 and < .50; large: ≥ .50) and explained variances R^2^ (small: ≥ .02 and < .13; medium: ≥ .13 and < .26; large: ≥ .26) referred to Cohen [36]. A two-tailed α of .05 was applied for evaluation of correlation and regression coefficients. Significant differences in correlation coefficients and explained variances R^2^ between WSSQ and WBIS as well as between the WSSQ subscales were examined by z tests for correlation coefficients with a one-tailed α of .05 using z-transformed correlation coefficients and R, respectively [37]. According to the conducted a-priori power analysis, *N* = 72 was necessary for detecting a large difference (effect size for correlation coefficients Cohen’s *q* = .50) between WSSQ and WBIS as well as between the WSSQ subscales with a power of .90. All analyses were conducted using the SPSS Statistical Software package version 20 (IBM Corp. Released 2011. IBM SPSS Statistics for Windows, Version 20.0. Armonk, NY: IBM Corp).

## Results

### Participants

As summarized in Table 1, the sample consisted of *N* = 78 prebariatric surgery patients with a mean age of *M* = 41.71 years (*SD* = 10.44) and a mean BMI of *M* = 48.86 kg/m^2^ (*SD* = 7.94). The sample showed moderate to high agreement with internalized weight stigma by reporting a mean WSSQ total score of *M* = 41.01 (*SD* = 7.42; range 21.00 to 56.73) and a mean WBIS score of *M* = 5.56 (*SD* = 1.02; range 2.00 to 7.00).

**Table 1:** Sample characteristics (*N* = 78).

	*M* (*SD*)	*n* (%)
Sex, female		55 (70.51)
Age, years	41.71 (10.44)	
BMI, kg/m^2^	48.86 (7.94)	
Weight status		
Class II obesity (35.0 ≤ BMI < 40.0 kg/m^2^)		7 (8.97)
Class III obesity (BMI ≥ 40.0 kg/m^2^)		71 (91.03)
WSSQ		
Total score	41.01 (7.42)	
Self-devaluation	19.73 (4.56)	
Fear of enacted stigma	21.38 (4.37)	
WBIS	5.56 (1.02)	
MBSRQ-AS		
Overweight preoccupation	3.61 (0.75)	
Appearance evaluation	1.44 (0.45)	
Appearance orientation	3.50 (0.71)	
IWQOL-Lite		
Total score	118.59 (23.00)	
Self-esteem	28.81 (6.27)	
PHQ-9	12.46 (5.36)	
GAD-7	7.96 (4.91)	

*Notes*. BMI, body mass index; WSSQ, Weight Self-Stigma Questionnaire–total score (12 to 60*, less favorable scores are asterisked); WSSQ–Self-devaluation/ Fear of enacted stigma (6 to 30*); WBIS, Weight Bias Internalization Scale (1 to 7*); MBSRQ-AS, Multidimensional Body-Self Relations Questionnaire–Appearance Scales–Overweight preoccupation (1 to 5*); MBSRQ-AS–Appearance evaluation (1* to 5); MBSRQ-AS–Appearance orientation (1 to 5*); IWQOL-Lite, Impact of Weight on Quality of Life–Lite Questionnaire–total score (31 to 155*; IWQOL-Lite self-esteem (7 to 35*); PHQ-9, Patient Health Questionnaire-depression scale (0 to 27*); GAD-7, 7-item Generalized Anxiety Disorder Scale (0 to 21*).

### Item Statistics of the WSSQ

The results with regard to the item statistics of the WSSQ are displayed in Table 2. In 17.95% of the sample at least one missing value was detected. Overall, the percentage of missing item responses was low with 2.67%, varying between 0% (items three and seven) and 10.26% (item one) of missing responses per item. Two items of the self-devaluation subscale (items one and five) yielded missing values > 5%. Distribution of all items deviated significantly from normality (*p*s < .05). While 10 of 12 items were negatively skewed (-0.93 ≤ skewness ≤ -0.14), only two items of each subscale (WSSQ self-devaluation: items two and three; WSSQ fear of enacted stigma: items seven and 11) differed significantly from normality with regard to skewness. Most items had a low kurtosis (-1.13 ≤ kurtosis ≤ +.26), however, only item one of the self-devaluation subscale differed significantly from normality with regard to kurtosis. The difficulty indices were of medium size (38 ≤ *p*_*m*_ ≤ 71), except for item 11 of the fear of enacted stigma subscale with high difficulty (*p*_*m*_ = 84). Corrected item-total-correlations were in the middle to upper range (.30 ≤ *r*_*it*_ ≤ .67) except for items one and two of the self-devaluation subscale with small corrected item-total-correlations (.15 ≤ *r*_*it*_ ≤ .25). Correlations between items and the corresponding subscale were in the medium to upper range (.47 ≤ *r* ≤ .75). All items were clearly assigned to the corresponding subscale as the correlations with the non-corresponding subscale were below *r* < .40 (-.03 ≤ *r* ≤ .36) except for item three of the self-devaluation subscale (*r* = .45) [38]. Altogether, both subscale scores were highly correlated with the total score (*r* = .82 to .83).

**Table 2:** Item statistics of the WSSQ (*N* = 78).

	*M*	*SD*	Skewness	Kurtosis	*p*_*m*_	*r*_*it*_	*r*_*self*_	*r*_*fear*_
1. I’ll always go back to being overweight	3.14	1.35	-.16	-1.13	54	.15	.48***	.03
2. I caused my weight problems	3.83	1.15	-.83	-.22	71	.25	.47***	-.03
3. I feel guilty because of my weight problems	3.74	1.18	-.80	-.11	69	.67	.65***	.45***
4. I became overweight because I’m a weak person	2.83	1.20	-.14	-1.06	46	.45	.72***	.27*
5. I would never have any problems with weight if I were stronger	2.51	1.25	.23	-1.08	38	.30	.48***	.27*
6. I don’t have enough self-control to maintain a healthy weight	3.51	1.29	-.43	-.99	63	.33	.69***	.11
7. I feel insecure about others’ opinions of me	3.82	1.13	-.93	.22	71	.45	.36**	.58***
8. People discriminate against me because I’ve had weight problems	3.47	1.25	-.42	-.83	62	.50	.26*	.75***
9. It’s difficult for people who haven’t had weight problems to relate to me	2.69	1.23	.21	-.78	42	.44	.23	.72***
10. Others will think I lack self-control because of my weight problems	3.77	1.01	-.53	-.43	69	.40	.25*	.52***
11. People think that I am to blame for my weight problems	4.35	0.72	-.86	.26	84	.35	.02	.58***
12. Others are ashamed to be around me because of my weight	3.22	1.19	-.30	-.69	56	.42	.17	.73***

*Notes*. WSSQ, Weight Self-Stigma Questionnaire; *p*_*m*_, item difficulty; *r*_*it*_, corrected item-total-correlation; *r*_*self*_, correlation with the WSSQ subscale self-devaluation; *r*_*fear*_, correlation with the WSSQ subscale fear of enacted stigma. Items one two six correspond to WSSQ self-devaluation, items seven to 12 to WSSQ fear of enacted stigma.

* p < .05

** p < .01

*** p < .001

### Internal Consistency of WSSQ and WBIS

Internal consistency in this study’s sample was acceptable for the total score of the WSSQ (α = .75) and its subscale fear of enacted stigma (α = .74), while the subscale self-devaluation yielded a questionable internal consistency (α = .63). Internal consistency of the WBIS was good (α = .84).

### Convergent Validity of WSSQ and WBIS

The results of the correlation analyses are displayed in Table 3. The WSSQ total score was significantly correlated with the WBIS (large effect). The WSSQ was positively associated with MBSRQ overweight preoccupation and appearance orientation (small effects), but not with MBSRQ appearance evaluation and BMI. In contrast, WBIS was correlated with MBSRQ appearance evaluation (medium effect) and appearance orientation (small effect), but not with MBSRQ overweight preoccupation and BMI. Overall, correlation coefficients did not differ between WSSQ and WBIS for any of the associations with MBQSR subscales.

**Table 3:** Correlations of the WSSQ and its subscales with validity measures and z test for differences in correlation coefficients.

	1	2	3	4	5	6	7	8	WSSQ vs. WBIS		WSSQ_self_ vs. WSSQ_fear_	
									*z*	*p*	*z*	*p*
1. WSSQ	1	-	-	*-*	-	-	-	-	-	-	0.13	.45
2. WBIS	.63***	1	-	-	-	-	-	-	-	-	1.86	.03
3. WSSQ self-devaluation	.83***	.42***	1	-	-	-	-	-	4.37	< .001	-	-
4. WSSQ fear of enacted stigma	.82***	.64***	.37**	1	-	-	-	-	2.46	.007	-	-
5. MBSRQ-AS overweight preoccupation	.29*	.13	.20	.25*	1	-	-	-	0.93	.18	0.27	.39
6. MBSRQ-AS appearance evaluation^a^	-.22	-.36**	-.13	-.30**	-.12	1	-	-	0.95	.17	1.07	.14
7. MBSRQ-AS appearance orientation	.23*	.29*	.16	.21	.27*	-.16	1	-	0.36	.36	0.33	.37
8. BMI	.20	.03	.12	.20	.08	-.23	-.21	1	1.06	.14	0.52	.30

*Notes*. WSSQ, Weight Self-Stigma Questionnaire; WBIS, Weight Bias Internalization Scale; WSSQ_self_, Weight Self-Stigma Questionnaire self-devaluation subscale; WSSQ_fear_, Weight Self-Stigma Questionnaire fear of enacted stigma subscale; MBSRQ-AS, Multidimensional Body-Self Relations Questionnaire–Appearance Scales; BMI, body mass index (kg/m^2^).

^*a*^ Spearman rank order correlations

* p < .05

**p < .01

***p < .001

Regarding WSSQ subscales, WSSQ self-devaluation was positively correlated with the WBIS (medium effect), but not with the MBSRQ scales and BMI. In contrast, WSSQ fear of enacted stigma was significantly associated with the WBIS, MBSRQ appearance evaluation (large effects), and overweight preoccupation (small effect), but not with MBSRQ appearance orientation and BMI. Correlation coefficients between WSSQ self-devaluation and WSSQ fear of enacted stigma differed significantly with respect to the WBIS.

In addition, WSSQ self-devaluation (*z*s = 0.46 to 0.52, *p*s = .30 to .32) and WSSQ fear of enacted stigma (*z*s = 0.02 to 0.55, *p*s = .29 to .49) did not differ from WSSQ total score with respect to explained variance of MBSRQ subscales and BMI. Correlation of the WSSQ total score with the WBIS was significantly higher than the association between WSSQ self-devaluation and WBIS (*z* = 1.75, *p* = .04).

### Prediction of Psychosocial Outcomes by WSSQ and WBIS

As summarized in Table 4, the WSSQ total score and the WBIS significantly predicted quality of life, self-esteem, depression, and anxiety with WSSQ predominantly yielding medium-size and WBIS predominantly yielding large-size effects. Specifically, the WBIS explained significantly more variance than the WSSQ total score for self-esteem. Regarding the WSSQ subscales displayed in Table 5, quality of life, self-esteem, and depression were significantly predicted by self-devaluation (small effects) and fear of enacted stigma (medium-to-large effects). Further, anxiety was significantly predicted by WSSQ fear of enacted stigma (small effect), but not by WSSQ self-devaluation. In particular, WSSQ fear of enacted stigma explained significantly more variance than WSSQ self-devaluation for quality of life and self-esteem.

**Table 4:** Linear regression analyses on psychosocial outcomes by measure of internalized weight stigma.

Dependent variable	Independent variable	B	SE	β	T	95% CI	R^2^	z	*p*
IWQOL-Lite total score	WSSQ	1.41	0.33	.45***	4.33	[0.76, 2.06]	.20	1.02	.15
	WBIS	12.86	2.11	.57***	6.10	[8.66, 17.07]	.33		
IWQOL-Lite self-esteem	WSSQ	0.43	0.09	.50***	5.01	[0.26, 0.60]	.25	2.86	.002
	WBIS	4.72	0.45	.77***	10.56	[3.83, 5.61]	.59		
PHQ-9	WSSQ	0.32	0.08	.43***	4.08	[0.16, 0.47]	.19	0.71	.24
	WBIS	2.82	0.53	.52***	5.31	[1.76, 3.88]	.27		
GAD-7	WSSQ	0.20	0.07	.30**	2.74	[0.06, 0.35]	.09	0.77	.22
	WBIS	1.99	0.50	.41***	3.97	[0.99, 2.98]	.17		

*Notes*. WSSQ, Weight Self-Stigma Questionnaire; WBIS, Weight Bias Internalization Scale; IWQOL-Lite, Impact of Weight on Quality of Life–Lite Questionnaire; PHQ-9, Patient Health Questionnaire–depression scale; GAD-7, 7-item Generalized Anxiety Disorder Scale.

* p < .05

**p < .01

***p < .001

**Table 5:** Linear regression analyses on psychosocial outcomes by subscale of the WSSQ.

Dependent variable	Independent variable	B	SE	β	T	95% CI	R^2^	z	*p*
IWQOL-Lite total score	WSSQ self-devaluation	1.34	0.58	.26*	2.32	[0.19, 2.49]	.07	1.84	.03
	WSSQ fear of enacted stigma	2.76	0.52	.52***	5.28	[1.71, 3.80]	.27		
IWQOL-Lite self-esteem	WSSQ self-devaluation	0.38	0.16	.27*	2.42	[0.07, 0.69]	.08	2.40	.008
	WSSQ fear of enacted stigma	0.85	0.13	.59***	6.35	[0.59, 1.12]	.35		
PHQ-9	WSSQ self-devaluation	0.38	0.13	.32**	2.83	[0.11, 0.64]	.10	0.53	.30
	WSSQ fear of enacted stigma	0.50	0.13	.40***	3.71	[0.23, 0.76]	.16		
GAD-7	WSSQ self-devaluation	0.20	0.12	.19	1.61	[-0.05, 0.45]	.03	0.90	.19
	WSSQ fear of enacted stigma	0.37	0.12	.32**	2.97	[0.12, 0.61]	.11		

*Notes*. WSSQ, Weight Self-Stigma Questionnaire; IWQOL-Lite, Impact of Weight on Quality of Life–Lite Questionnaire; PHQ-9, Patient Health Questionnaire–depression scale; GAD-7, 7-item Generalized Anxiety Disorder Scale.

* p < .05

**p < .01

***p < .001

It is further noteworthy that the WSSQ self-devaluation (*z*s = 0.75 to 1.63, *p*s = .05 to .23) as well as the WSSQ fear of enacted stigma (*z*s = 0.14 to 0.76, *p*s = .22 to .44) did not differ from the WSSQ total score with respect to explained variance of quality of life, self-esteem, depression, and anxiety.

## Discussion

The present study is the first to present item statistics of the German version of the WSSQ. Further, it is unique in providing and comparing reliability, convergent validity with and predictive values for psychosocial health outcomes for two self-report measures on internalized weight stigma, the WSSQ and the WBIS. Altogether, the results suggest the WSSQ and the WBIS to be reliable and valid assessments of internalized weight stigma in prebariatric surgery patients. Given the lack of studies comparing the WSSQ and the WBIS, the present study provides initial evidence for the comparability of both self-report questionnaires to assess internalized weight stigma in prebariatric surgery patients: both measures did not differ with respect to overall convergent validity with and predictive values for multiple psychosocial health outcomes, while findings indicate better internal consistency of the WBIS than that of the WSSQ.

Regarding item statistics of the WSSQ, overall, a low number of missing data was found, although two items of WSSQ self-devaluation yielded a relatively large amount of missing values (> 5%) which may bias statistical analyses of this subscale [39]. As expected [14], prebariatric surgery patients showed moderate to high agreement with internalized weight stigma on most items resulting in items’ significant deviation from normality with mostly flat distributions (low kurtosis) with a long tail to the left (negative skew). Predominantly, medium item difficulties and favorable positive corrected item-total correlations were detected indicating that items of the WSSQ are appropriate to differentiate between individuals with high versus low internalized weight stigma. Based on correlation coefficients with the corresponding and non-corresponding subscale, results might support the two-factor solution of the WSSQ, although one item of WSSQ self-devaluation could not be clearly assigned to the corresponding subscale. Altogether, findings provide evidence for predominantly favorable item statistics of the German version of the WSSQ.

Results further suggest the WSSQ and the WBIS to be reliable assessments of internalized weight stigma in prebariatric surgery patients, although the results indicate internal consistency of the WSSQ to be less than expected and slightly less than that of the WBIS. The present findings are in line with a recently published study which was not available at time of study conduct [16] and provided first evidence for good reliability of a German version of the WSSQ in individuals with at least obesity class II, although internal consistency for the WSSQ total score was slightly higher compared to internal consistency in the present study. Moreover, the original validation study of the WSSQ reported even larger internal consistency in individuals with overweight and obesity [1]. Relatedly, internal consistency of the 10-item version of WBIS in the present study was comparable to that of a previous study in prebariatric surgery patients [14], but slightly smaller compared to previous research in individuals with overweight and obesity [15]. Yet, it is difficult to compare findings between bariatric surgery samples and individuals with overweight and obesity in general as bariatric surgery candidates are a specific subsample of this population with respect to multiple health outcomes including internalized weight stigma [14,15,40]. Potentially, internalized weight stigma is a more homogeneous construct in non-bariatric samples compared to bariatric surgery samples.

Regarding convergent validity, hypotheses were confirmed as the WSSQ and the WBIS were positively associated with each other and significantly associated with negative body image. Adding to previous research that produced mixed findings [1,2,16], the WSSQ and the WBIS were not associated with BMI suggesting that public weight stigma was internalized irrespective of individuals’ relative weight. Both measures did not differ with respect to convergent validity with different body image aspects and BMI. As a specific analysis of convergent validity, the WSSQ and the WBIS cross-sectionally predicted poorer quality of life and self-esteem as well as greater depression and anxiety, which was conform to our hypotheses. Predictive values for these psychosocial health outcomes did not differ between WSSQ and WBIS except for self-esteem with the WBIS showing greater predictive power. Findings are in line with previous research providing evidence for good convergent validity of the WSSQ, for instance with psychological distress, quality of life, weight stigma, depression, eating behavior, body image, dissociative symptoms [1,16], and the WBIS [15].

Regarding the WSSQ subscales, reliability was less than expected especially for the WSSQ subscale self-devaluation. However, findings are in line with the original validation study providing first evidence for good convergent validity of both subscales [1]. Overall, results indicated superiority of the WSSQ subscale fear of enacted stigma compared to the subscale self-devaluation with respect to item statistics, reliability, convergent validity, and predictive values, which might be due to greater overlap with the WBIS as the results showed. Based on the fact that WSSQ subscales showed reduced reliability and absent incremental values with regard to convergent validity with and predictive values for psychosocial health outcomes compared to the WSSQ total score, research and clinical practice may rather administer the WSSQ total score than its subscales.

Our findings need to be interpreted while taking into account the strengths and limitations of this study. Strengths include the blinded back-translation of the WSSQ by a licensed translator. Further, the present study used other internationally well-established self-report questionnaires and other constructs for validation of the WSSQ that were often not assessed in previous studies, i.e., the WBIS as alternative assessment of internalized weight stigma, diverse body image aspects (MBSRQ-AS), anxiety (GAD-7), and depression (PHQ-9) [1,16]. In addition, social desirability of ratings was reduced– even if not eliminated –by informing patients that data collection would be unrelated to psychological evaluation prior to surgery and the information obtained would not be shared with the clinicians. However, face-validity of the translated WSSQ was evaluated only by a research team with extensive expertise in the field of obesity instead of a pre-test with individuals with overweight and obesity, which clearly presents a limitation of this study’s findings. Further, internal consistencies of MBSRQ overweight preoccupation and MBSRQ appearance evaluation applied for evaluation of convergent validity were poor to unacceptable. This might be due to the small number of items (four to seven) as well as to the special sample consisting of bariatric surgery candidates, in which body image was not suggested to be a homogeneous construct. Interpretation of our results is further limited due to the small sample size, which restricts findings’ generalizability on the prebariatric surgery population. It further limits clear interpretation of item statistics and prohibits factor analysis of the WSSQ because of too small communalities (h^2^ < .60) [41]. Cross-sectional data prevent firm conclusions on predictive validity.

As a consequence, future studies need to compare WSSQ and WBIS in a larger bariatric surgery sample, which would also offer the opportunity for validation and to examine the postulated factor structure of the WSSQ. In this context, individuals undergoing bariatric surgery are of special interest as they report particularly high internalized weight stigma [14,15]. Further, they are a specific subsample of the population with obesity differing not only in weight status but also reporting less favorable values in several psychosocial aspects after adjusting for BMI, for example, in physical and social functioning [40]. Hence, differences between WSSQ and WBIS could be compared between a bariatric surgery and a large general population sample with overweight and obesity providing as well population norms for the WSSQ. In addition, longitudinal studies are needed in order to provide data about stability and predictive validity of both measures, for example, for weight-related and psychosocial outcomes.

In conclusion, our findings provide first evidence for reliable and valid assessment of internalized weight stigma in prebariatric surgery patients by the WSSQ and the WBIS. This is of importance for improving treatment outcomes in clinical practice, as individuals with high internalized weight stigma–in bariatric as well as conservative treatment settings–might be at high risk for greater psychosocial impairment and smaller weight loss [2,14,15,17–25]. Clinically, the WSSQ and the WBIS could be used as screening instruments for identifying those who would benefit from specialized interventions aiming at the reduction of internalized weight stigma, i.e, by psychoeducation, body image interventions, cognitive restructuring of negative body talk, and detaching self-evaluation from stereotypes [42]. However, as long as validation studies on the WSSQ providing population norms are still missing, clinical practice and research might give preference to the WBIS in bariatric surgery samples, because of the marginally better reliability, convergent validity, and greater predictive power.

## Supporting Information

S1 Data Supporting Data. (SAV)
